# The Anti-Inflammatory Properties of Chaga Extracts Obtained by Different Extraction Methods against LPS-Induced RAW 264.7

**DOI:** 10.3390/molecules27134207

**Published:** 2022-06-30

**Authors:** Weaam Alhallaf, Lewis B. Perkins

**Affiliations:** 1 School of Food & Agriculture, Rogers Hall, University of Maine, Orono, ME 04469, USA; weaam.al@maine.edu; 2 College of Education for Women, University of Baghdad, Baghdad 17001, Iraq

**Keywords:** natural product extracts, nutraceuticals, inflammation treatment, healthful fungal products

## Abstract

Chaga, a sclerotia formed by the *Inonotus obliquus* fungus, has been widely recognized for a number of medicinal properties. Although numerous scientific investigations have been published describing various biological activities of chaga from different geographical locations, little work has focused on chaga harvested in the USA or extraction techniques to maximize anti-inflammatory properties. The aim of this study was to investigate the anti-inflammatory properties of chaga collected in Maine (USA) extracted using traditional aqueous (hot water steeping) methods against lipopolysaccharide (LPS)-induced RAW 264.7 macrophages. Chaga extracts obtained from both conventional (ethanol/water) extraction methods and an accelerated solvent extraction method (ASE) at optimized conditions were compared to aqueous extracts (tea) obtained from chaga in the powder form (P) and powder form in tea bags (B) based on their effect on both nitric oxide (NO) production and pro-inflammatory cytokine expression, in particular, the expression of TNF-α, interleukin-6 (IL-6), and interleukin-β (IL-1β). Phenolic acid extracts from chaga and individual phenolic acid standards were also investigated for their effect on the same parameters. Results indicated that various chaga extracts have significant anti-inflammatory activity on LPS-stimulated RAW 264.7 cells. The inhibitory effect was through a decrease in the production of NO and the downregulation of TNF-α, IL-6, and IL-1β in RAW 264.7 macrophages. ASE1 (novel, optimized ethanol/water extraction) and P6 (six-minute steeping of powder in 100 °C water) extracts showed the highest inhibitory activity on NO production and on the expression of the inflammatory cytokines, compared to extracts obtained by conventional extraction methods.

## 1. Introduction

Inflammation is a physiological immune response of the body to injury, characterized by fever, swelling, and pain, and is usually implicated in the pathogenesis of a variety of diseases including, asthma, heart disease, cancer, and diabetes [1] During the inflammatory process, large amounts of pro-inflammatory mediators such as nitric oxide (NO), prostaglandin E2 (PGE2), tumor necrosis factor-α (TNF-α), interleukin-6 (IL-6), and interleukin-1β (IL-1β) are generated, mostly for the primary protection of the host [2,3]. However, excess uncontrolled production of these inflammatory products can lead to oxidative stress [3,4]. Recently, there has been considerable interest in finding anti-inflammatory agents with low toxicity, from natural sources [1]. Phenolic compounds are among the many natural substances extracted from plant and fungal sources attracting attention for anti-inflammatory and other biological activities.

Previous studies have shown that chaga (*Inonotus obliquus*) contains various bioactive components with a range of chemical characteristics and polarities [5,6,7]. Accordingly, extraction of bioactive compounds from chaga requires various solvents targeted to specific chemical profiles. For example, petroleum ether and chloroform are used to extract lanostane-type triterpenoids from chaga; whereas water and aqueous alcohol are suitable solvents targeted for polysaccharides, melanin pigments, and phenolic compounds [8,9]. Aqueous preparations of chaga have been used since the 12th century in Eastern Europe to treat a variety of ailments without toxic effect. The traditional preparation method for chaga tea is steep or boil the sclerotia (small pieces or powder) in water for a few hours or a few days. The resultant extracts from these processes can be immediately consumed or stored at a suitable temperature [10]. Currently, “quick brewing” is a more common way to consume chaga, most often as a tea. Chaga in powder form, usually in tea bags, is steeped in hot water (80–100 °C) for a several minutes, strained, and then consumed as a tea. Investigations of the chemical composition and biological properties of aqueous extracts from chaga have shown therapeutic effects against diabetes via multiple pathways, including antioxidative [11]. Research has found that orally administered aqueous extract of chaga can ameliorate acute inflammation [12]. However, no investigation has been carried out to examine the chemical composition of chaga tea in powder and tea-bag form.

Alcoholic extracts from chaga are characterized by high phenolic content. It has been reported that phenolics are the main chemical compounds involved in chaga’s biological effects—including its antioxidant, anti-cancerous, antimicrobial, and anti-inflammatory activities [13,14,15,16,17,18,19]. A variety of conventional and advanced extraction methods have been used to isolate phenolic compounds from chaga [20,21]. Recent work [22] found that accelerated solvent extraction (ASE) increases the total phenolic content and enhances the DPPH-scavenging activity of chaga extracts, as compared to conventional extraction methods. In addition, the concentrations of individual phenolic acids significantly increased compared to concentrations in other extracts. Previous studies have demonstrated that alcoholic extracts from chaga possess significant anti-inflammatory effects in vivo and in vitro. However, no studies have investigated the effect of the extraction method on the anti-inflammatory properties of chaga extracts.

The focus of the work presented in this manuscript is the investigation of the effect of chaga extracts obtained from a number of consumer-based and laboratory extraction techniques on anti-inflammation in LPS-stimulated 264.7 cells. Extraction methods include powdered (P3, P6, P10) and bagged powdered (B3, B6, B10) chaga steeped in 100 °C water for 3, 6, and 10 min. Chemical analysis was performed on these aqueous-based extracts. Traditional extraction in 70/30 ethanol/water (*v*/*v*) using maceration (ME), soxhlet (SE), reflux (RE); and accelerated solvent extraction (ASE) at 130 (1), 150 (2) and 170 °C (3) were also tested on the aforementioned anti-inflammatory cell model. This is the first research focused on birch chaga collected in Maine, USA and also examines the biologically active chemical features of chaga tea steeping in bag and powder form.

## 2. Results

### 2.1. Chemical Composition

The chemical yield and the bioactive content of the crude extracts from the powdered form of chaga were significantly higher (*p* < 0.05) than those of the bagged form at all extraction temperatures (Table 1). For example, P6 yielded 30.21 ± 0.01% crude polysaccharide containing 17.56 ± 0.01% carbohydrate, 10.23 ± 1.02% protein, 4.12 ± 0.44% uronic acid, and 17.61 ± 0.05% (*w*/*w*) phenolic content, while B6 yielded 17.33 ± 0.03% crude polysaccharide containing 11.56 ± 0.01% carbohydrate, 7.11 ± 0.5% protein, 2.57 ± 0.71% uronic acid, and 8.33 ± 0.05% (*w*/*w*) phenolic content. The results also indicated that brewing time had no significant effect on the yield and the chemical content of the crude extracts of both bagged and powdered form. The B3 extract resulted in a carbohydrate content of 10.26 ± 0.08% (*w*/*w*), which was comparable to the carbohydrate content of 11.56 ± 0.01% and 11.81 ± 0.04% (*w*/*w*) obtained from B6 and B10 extracts, respectively. Similarly, P3, P6, and P10 extracts had carbohydrate content of 17.02 ± 0.01%, 17.56 ± 0.01%, and 18.31 ± 0.05% (*w*/*w*), respectively. The same trends were observed for the yield, protein, uronic acid, and total phenolic content of crude polysaccharide extracts of chaga tea obtained from different brewing times.

### 2.2. Cell Viability

**Figure 1:**
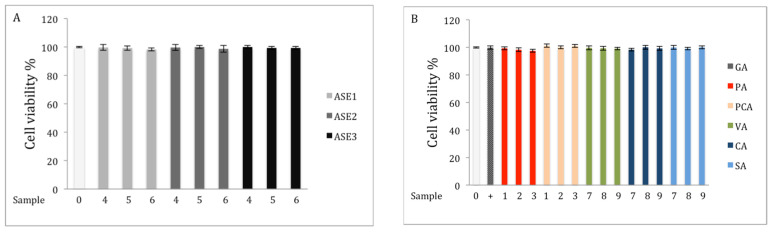

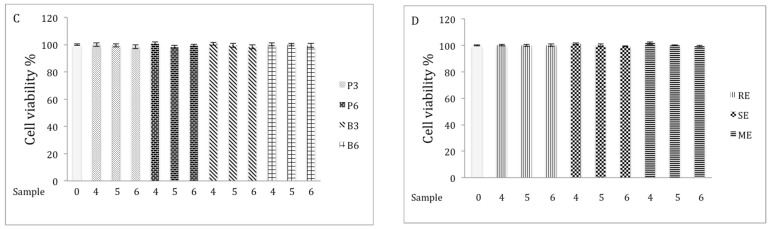
Effects of different extracts and phenolics on cell viability of RAW264.7 cells. Extracts were obtained by (**A**) ASE conditions, (**B**) pure phenolic acid standards, (**C**) crude polysaccharide extracts, and (**D**) different extraction methods. Cells were stimulated with 1 µg/mL of LPS plus varying concentrations of samples (0 = media, GA = 2 μM; 1 = 5 μM; 2 = 10 μM; 3 = 20 μM; 4 = 50 μg/mL; 5 = 100 μg/mL; 6 = 150 μg/mL; 7= 25 μM; 8 = 50 μM; and 9 = 100 μM).

RAW 264.7 cells were treated with various chaga extracts or pure phenolic standards: 50, 100, and 150 μg/mL chaga extracts; or 25, 50, and 100 μM vanillic acid (VA), caffeic acid (CA), or syringic acid (SA); or 5, 10, and 20 μM protocatechuic acid (PA) or protocatechuic aldehyde (PCA) to assess their effects on cell viability using the MTT (3-(4,5-dimethylthiazolyl-2)-2,5-diphenyltetrazolium bromide) assay. This assay measures the activity of the mitochondrial succinate-tetrazolium reductase of living cells and its ability to cleave the tetrazolium salts to formazan crystals, resulting in a color change that can be monitored spectrophotometrically [23]. The data were expressed as percent cell viability compared t*o* control (0.05% DMSO). The results revealed that chaga extracts and the pure standards caused no cytotoxicity at the examined concentrations in RAW 264.7 cells (Figure 1) and subsequent experiments were performed at these concentrations.

### 2.3. Inhibition of NO Production in LPS-Stimulated RAW 264.7 Macrophages

**Figure 2:**
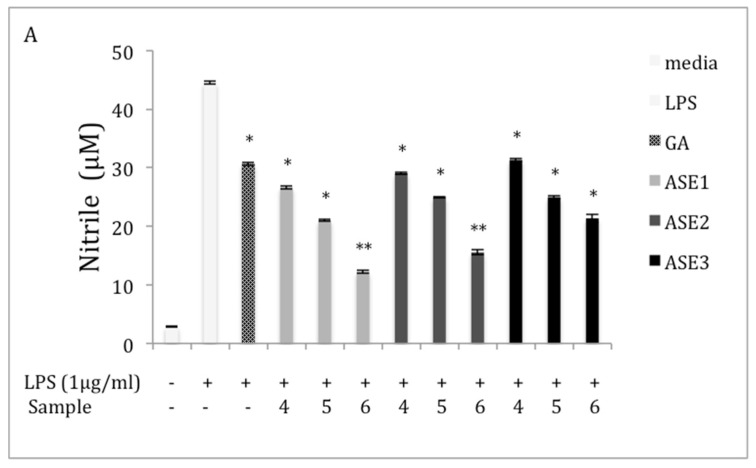

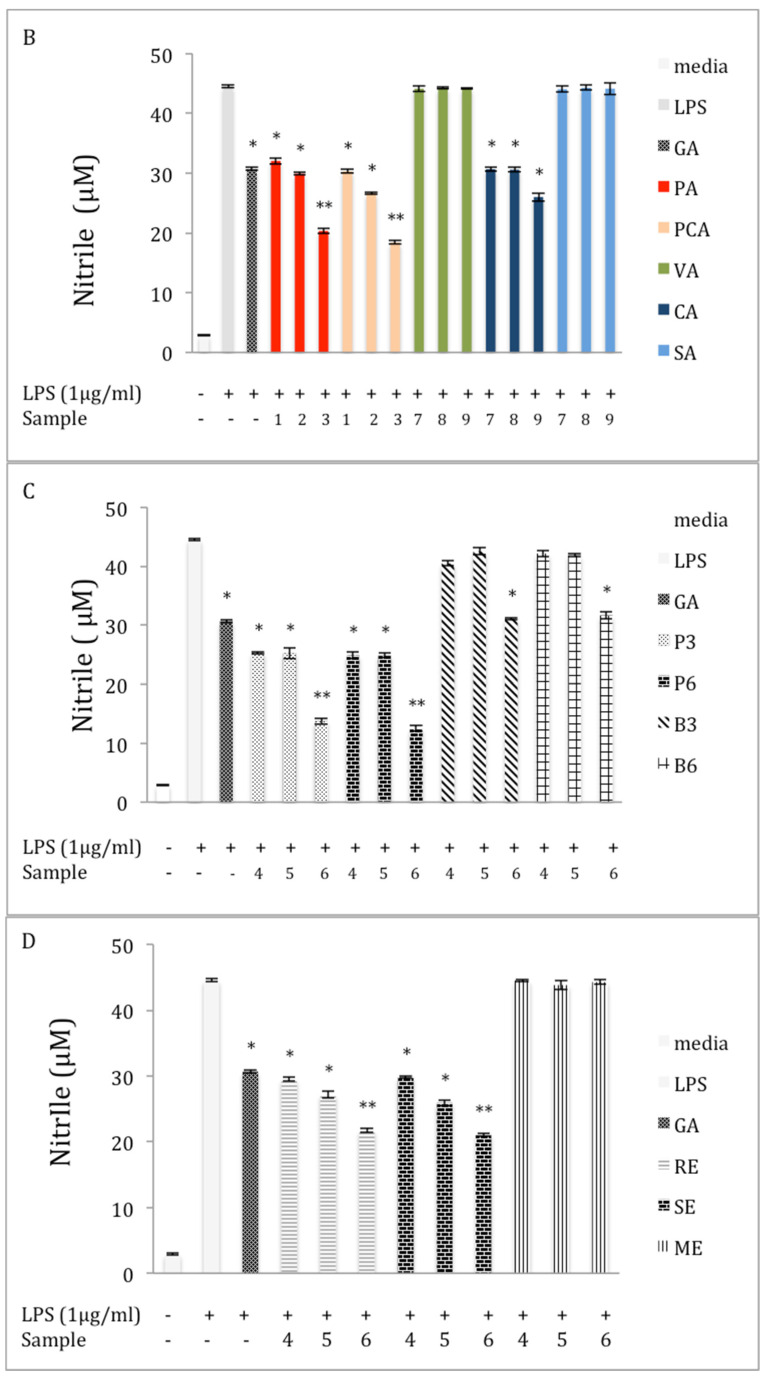
Effect of extracts and phenolics on production of nitric oxide (NO) in macrophage RAW 264.7 cells (**A**) ASE extracts; (**B**) pure phenolic standards; (**C**) crude polysaccharide extracts; and (**D**) different extraction methods. Cells were cultured in the absence (−) or presence (+) of LPS (1 μg/mL) with various concentrations of different samples (- denotes no sample) for 24 h (0 = media; GA = 2 μM; 1 = 5 μM; 2 = 10 μM; 3 = 20 μM; 4 = 50 μg/mL; 5 = 100 μg/mL; and 6= 150 μg/mL; 7 = 25 μM; 8 = 50 μM; and 9 = 100 μM). NO production was measured by the Griess reagent and is represented as mean ± standard error (SE) in the bars. Statistical significance *p* < 0.05 (*) and *p* < 0.01 (**) was determined using one-way analysis of variance for independent means, followed by Tukey’s HSD test.

Inhibitory effects of various chaga extracts and pure phenolic acid standards on NO production on LPS-induced RAW 264.7 cells were examined. Stimulation of the cells with 1 μg/mL LPS increased the NO levels to 44.53 ± 0.23 μM as compared to 4.8 ± 0.12 μM in the negative control group (Figure 2). All ASE extracts significantly (*p* < 0.05) reduced the level of NO production at all tested concentrations (Figure 2A). It is of note that at 150 μg/mL, ASE1, ASE2, and ASE3 reduced the concentration of NO released from RAW 264.7 cells by 66.82%, 61.61%, and 43.34%, respectively, compared with the LPS group. We further investigated the inhibitory effect of the main phenolic acids found in chaga extracts on the inhibition of NO production. Results showed that treating the LPS-induced cells with various concentrations of PA, PCA, and CA significantly (*p* < 0.05) reduced the production of NO, while treatments with SA and VA did not exhibit any effect on the induced cells (Figure 2B). At the highest examined concentrations, the levels of NO released from the induced cells decreased by 54.35%, 58.66%, and 41.60% after treatments with PA, PCA, and CA, respectively. However, the VA and SA treatments did alter the level of NO production at all concentrations.

All crude polysaccharide extracts separated from chaga tea in the powder form showed a significant inhibitory effect (*p* < 0.05) on NO production (Figure 2C). However, crude polysaccharide extracts separated from the bag form of chaga tea exhibited a significant inhibitory effect (*p* < 0.05) on NO production only at the highest examined concentration of the extracts compared to the LPS group. For example, at 150 μg/mL, P6 and B6 extracts reduced the nitrile concentration in the supernatants by 67.76% and 37.31%, respectively, compared to the LPS group.

To investigate the effect of the conventional extraction methods on the inhibition of NO production, RAW264.7 cells were incubated with extracts obtained by different extraction methods in the presence of 1 μg/mL of LPS. ME had no effect on the inhibition of NO production, but both RE and SE extracts showed significant inhibition (*p* < 0.05) on the production of NO, compared to the LPS group (Figure 2D). At 150 μg/mL, RE and SE extracts reduced the nitrile concentration in the supernatants by 51.42% and 52.61%, respectively, compared to the LPS group.

### 2.4. Inhibition of TNF-α Production in LPS-Stimulated RAW 264.7 Macrophages

**Figure 3:**
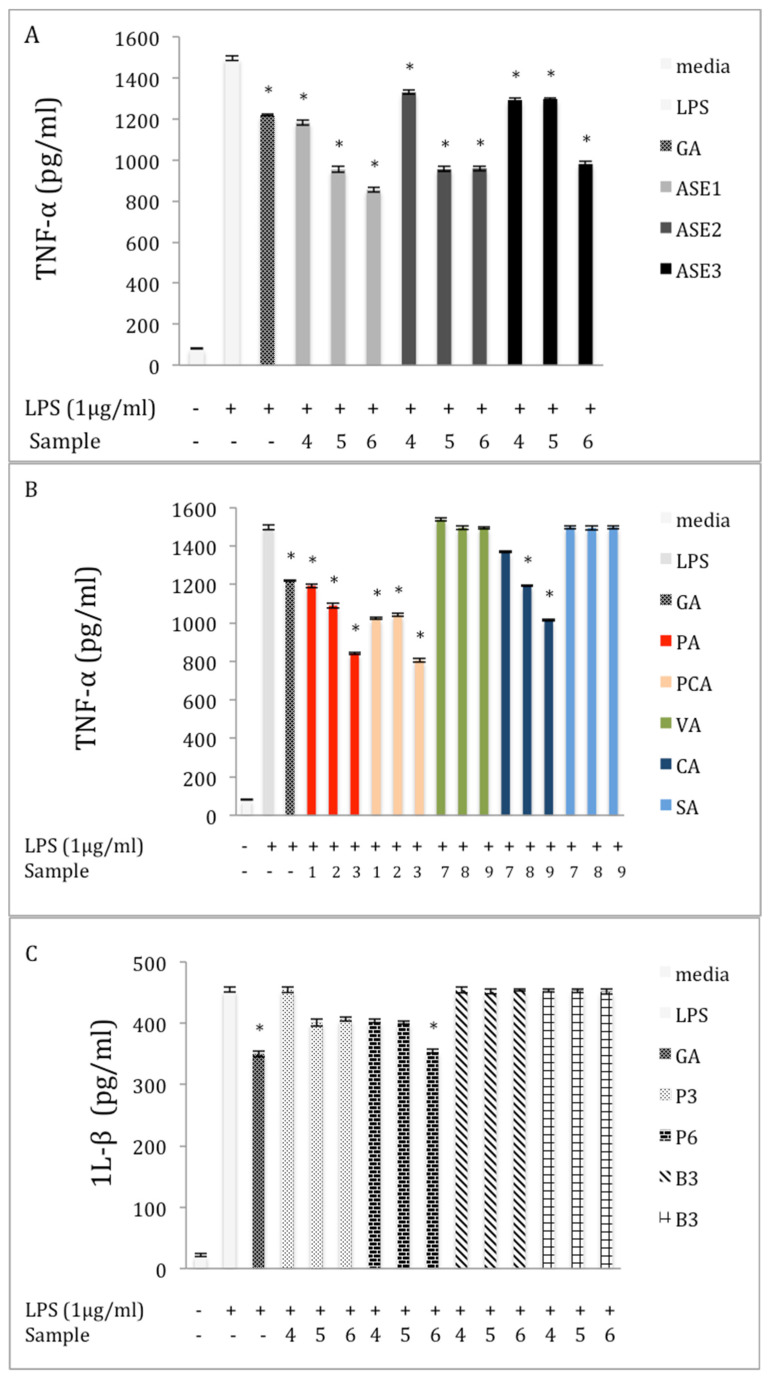

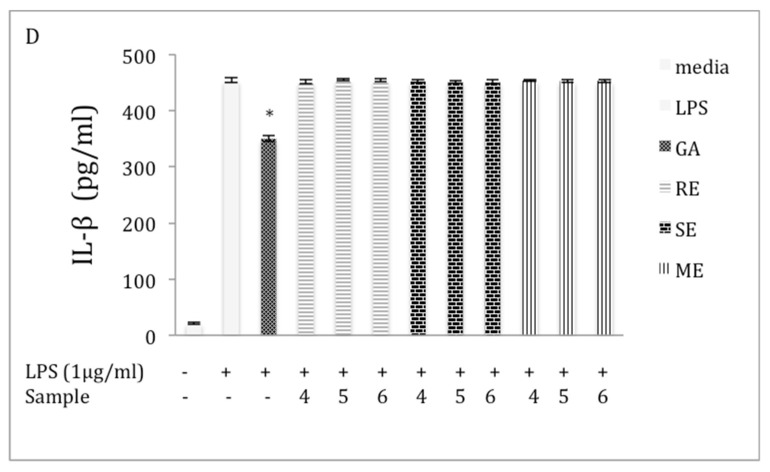
Effect of extracts and phenolics on tumor necrosis factor-α (TNF-α) expression in macrophage RAW 264.7 cells (**A**) ASE extracts; (**B**) pure phenolic standards; (**C**) crude polysaccharide extracts; and (**D**) different extraction methods. Cells were cultured in the absence or presence of LPS (1 μg/mL) with various concentrations of different samples for 24 h (0 = media; GA = 2 μM; 1 = 5 μM; 2 = 10 μM; 3 = 20 μM; 4 = 50 μg/mL; 5 = 100 μg/mL; and 6 = 150 μg/mL; 7 = 25 μM; 8 = 50 μM; and 9 = 100 μM). TNF-α production was determined through an ELISA. The data represent the mean ± SE of triplicate experiments. Statistical significance *p* < 0.05 (*) was determined using one-way analysis of variance for independent means, followed by Tukey’s HSD test.

To evaluate the anti-inflammatory effect of chaga, the inhibitory effects of various extracts and pure phenolic standards on the expression of the pro-inflammatory cytokines TNF-α, 1L-6, and IL-1β in LPS-stimulated RAW 264.7 cells were investigated. We found an increase in the expression of TNF-α in the LPS stimulation group compared to the control group (Figure 3). Twenty-four hours of incubation with ASE extracts significantly inhibited the level of TNF-α (*p* < 0.05) in the LPS-induced cells, compared with the LPS group. At a concentration of 150 μg/mL, ASE1, ASE2, and ASE3 extracts reduced the level of TNF-α released from RAW 264.7 cells by 42.90%, 35.94%, and 31.15%, respectively, as compared to the LPS group (Figure 3A). The effect of the pure phenolic standards on the expression of TNF-α is presented in Figure 3B. All phenolic acids except VA and SA significantly (*p* < 0.05) decreased the secretion of TNF-α at all examined concentrations, compared to the LPS treatment. At the highest concentrations, PA, PCA, and CA reduced the expression of TNF-α by 44.3%, 46.7%, and 32.5%, respectively, compared to the LPS treatment. The results from the crude polysaccharide extracts showed that extracts from the powder form of chaga tea had significant inhibitory effects (*p* < 0.05) on the level of TNF-α, compared with the LPS group. At a concentration of 150 μg/mL, P3 and P6 extracts reduced the level of TNF-α in the supernatants by 37.2% and 37.5%, respectively (Figure 3C).

The level of NO inhibited by chaga extracts depended on extraction method. Further examination found that extraction method affected the attenuation level of TNF-α. At a concentration of 150 μg/mL, extracts made by RE and SE had significant effects (*p* < 0.05) on the level of TNF-α, compared to LPS treatment. For example, TNF-α expression was inhibited by 22.3%, and 22.1% after treatment with 150 μg/mL of RE, and SE, respectively. The results also showed that ME extract had no inhibitory effect on the level of TNF-α at any concentration (Figure 3D).

### 2.5. Inhibition of IL-6 Production in LPS-Stimulated RAW 264.7 Macrophages

**Figure 4:**
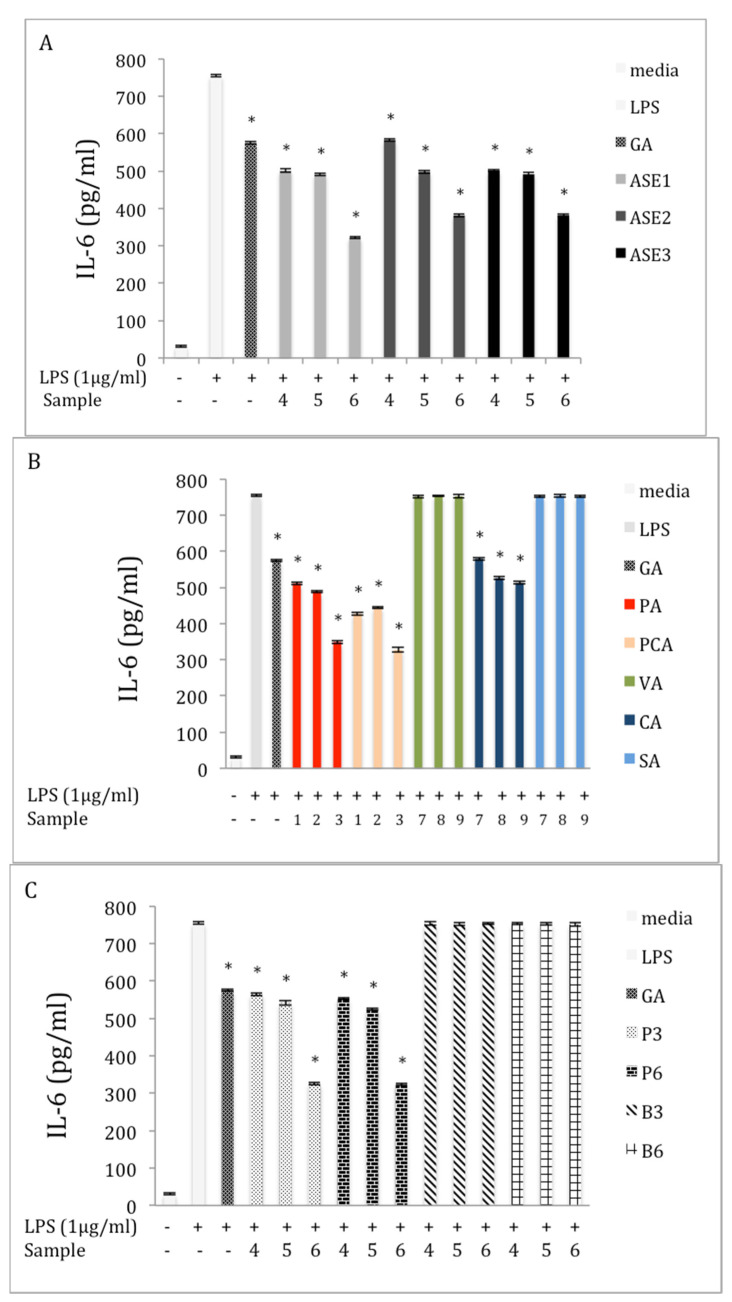

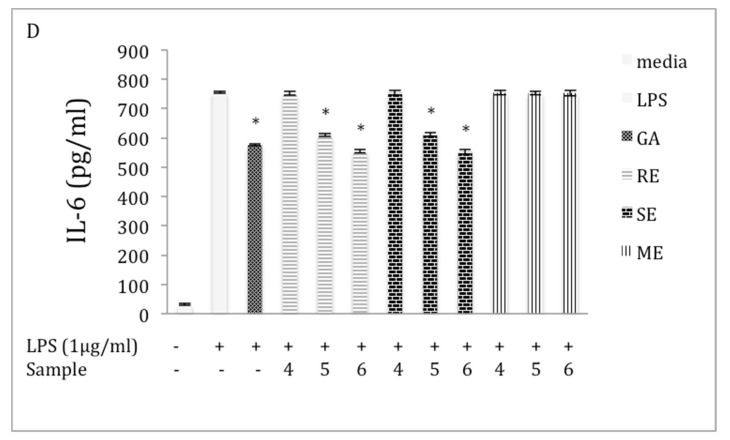
Effect of extracts and phenolics on IL-6 expression in macrophage RAW 264.7 cells (**A**) extracts obtained by ASE; (**B**) pure phenolic acid standards; (**C**) crude polysaccharide extracts; and (**D**) different extraction methods. Cells were cultured in the absence or presence of LPS (1 μg /mL) with various concentrations of different samples for 24 h (0 = media; GA = 2 μM; 1 = 5 μM; 2 = 10 μM; 3 = 20 μM; 4 = 50 μg/mL; 5 = 100 μg/mL; and 6 = 150 μg/mL; 7 = 25 μM; 8 = 50 μM; and 9 = 100 μM). IL-6 production was determined through an ELISA. The data represent the mean ± SD of triplicate experiments. Statistical significance *p* < 0.05 (*) was determined using one-way analysis of variance for independent means, followed by Tukey’s HSD test.

The expression of IL-6 cytokine increased to (755 ± 0.42 pg/mL) in macrophage cells after stimulation with LPS. However, when various chaga extracts obtained by ASE were added at 50, 100, and 150 μg/mL, these increases were significantly (*p* < 0.05) reduced. For example, at 150 μg/mL, ASE1, ASE2, and ASE3 extracts reduced the level of IL-6 released from RAW 264.7 cells by 57.3%, 49.4%, and 50.4%, respectively, compared with the LPS group (Figure 4A). The inhibition activity of phenolic acid standards on the expression of IL-6 in LPS-induced 264.7 RAW cells is illustrated in Figure 4B. The same trend of TNF-α expression inhibition for all levels of IL-6 was observed for all assayed phenolic acids except VA and SA (*p* < 0.05). The levels of IL-6 at all examined concentrations were suppressed compared to the LPS treatment. At the highest concentrations, PA, PCA, and CA reduced the expression of IL-6 by 53.7%, 56.6%, and 39.5%, respectively, compared to the LPS treatment. The effect of the polysaccharide extracts on the expression of IL-6 was similar to their effect on TNF-α; the expression of IL-6 was significantly reduced (*p* < 0.05) after treating the induced cells with P3 and P6 extracts at different concentrations. For example, the IL-6 level was reduced by 56.8% and 57.1% after the LPS-induced cells were treated with 150 μg/mL of P3 and concentration (Figure 4C). Figure 4D shows that extracts obtained by SE and RE had a significant inhibitory effect (*p* < 0.05) on the expression of IL-6 cytokine compared with the LPS group. At a concentration of 150 μg/mL, RE and SE inhibited the cytokine level by 26.6% and 26.9%, respectively, compared with the LPS group. No inhibitory effect of ME extract on the level of IL-6 was observed at any concentration.

### 2.6. Inhibition of IL-β Production in LPS-Stimulated RAW 264.7 Macrophages

**Figure 5:**
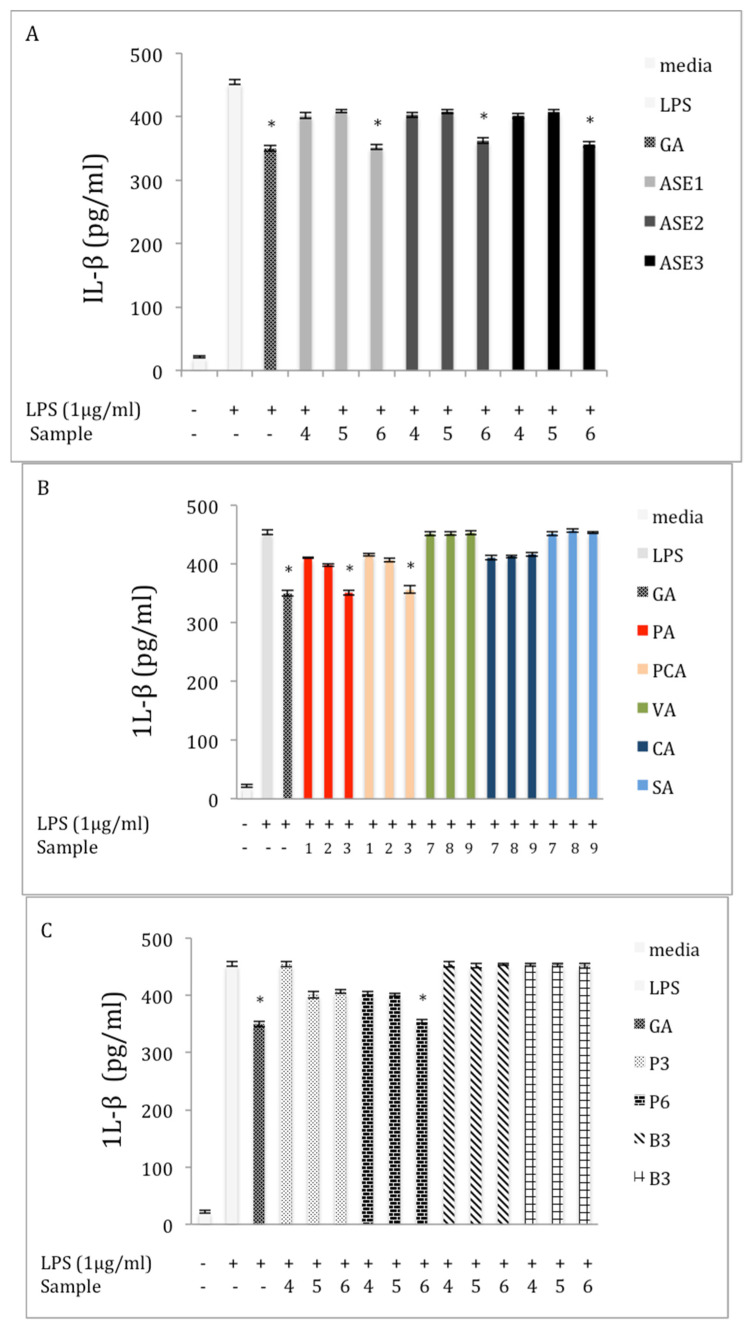

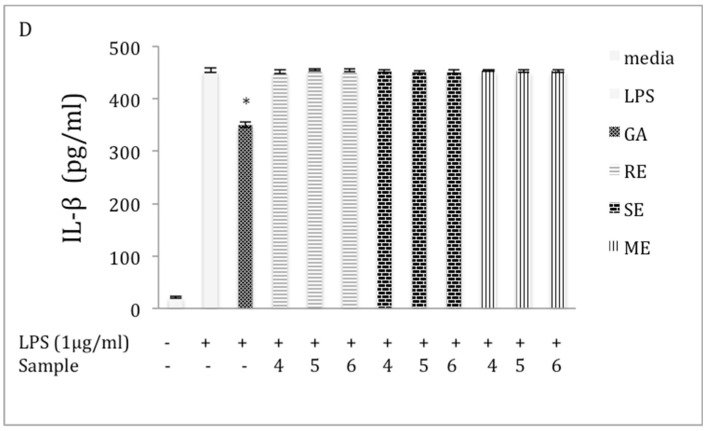
Effect of extracts and phenolics on IL-β expression in macrophage RAW 264.7 cells (**A**) extracts obtained by ASE; (**B**) pure phenolic acid standards; (**C**) crude polysaccharide extracts; and (**D**) different extraction methods. Cells were cultured in the absence or presence of LPS (1 μg /mL) with various concentrations of different samples for 24 h (0 = media; GA = 2 μM; 1 = 5 μM; 2 = 10 μM; 3 = 20 μM; 4 = 50 μg/mL; 5 = 100 μg/mL; and 6 = 150 μg/mL; 7 = 25 μM; 8 = 50 μM; and 9 = 100 μM). IL-β production was determined through an ELISA. The data represent the mean ± SD of triplicate experiments. Statistical significance *p* < 0.05 (*) was determined using one-way analysis of variance for independent means, followed by Tukey’s HSD test.

Our data indicated an increase in the expression of IL-β in the LPS-stimulation group compared to the control group (Figure 5). At 150 μg/mL, all chaga extracts obtained by ASE displayed a significant inhibitory effect (*p* < 0.05) on the level of IL-1β as compared to the LPS group (Figure 5A). For example, ASE1 reduced the level of IL-1β by 22.6% compared to the LPS group. Similarly, the level of IL-1β decreased significantly (*p* < 0.05) after treating the LPS-induced cells with the highest concentrations of PA and PCA, respectively (Figure 5B). For example, the concentration of IL-1β in the supernatants was decreased by 22.6% and 21.5%, after incubation of the LPS-induced cells with highest concentration of PA and PCA, respectively. The effect of the polysaccharide extracts on the expression of IL-β is presented in Figure 5C. Polysaccharide extracts from the chaga obtained from the tea-bag form did not affect the level of inflammatory cytokine. However, at 150 μg/mL, P6 significantly (*p* < 0.05) reduced the level of IL-1β, by 21.5%, compared to the LPS treatment. Figure 5D presents the effect of the extraction method on the inhibition activity of the extracts against IL-1β in the LPS-induced macrophages. None of the extracts obtained by the described conventional methods affected the expression of the IL-1β at any concentration compared to the LPS group.

## 3. Discussion

Inflammation is a physiological immune response of body tissues against physical, chemical, and biological stimuli such as tissue injury, chemical toxins, or pathogens [3]. Lipopolysaccharide, an endotoxin, is an integral outer membrane component of gram-negative bacteria, and the most potent trigger for microbial initiators of inflammatory response [24]. Macrophages play a vital role in the immune system and are associated with inflammatory diseases as initiators of pathogen- or tissue-induced inflammation. Macrophages activated by LPS treatment produce a wide variety of inflammatory markers, including NO TNF-α, IL-6, and IL-1β, primarily for the protection of the host. However, excess and uncontrolled production of inflammatory products often leads to excessive inflammatory response and oxidative stress [4,25,26]. Anti-inflammatory agents produce an anti-inflammatory effect through regulating cytokines and these inflammatory mediators. Therefore, monitoring the expression of these mediators is vital for understanding the inflammatory process and provides a measure to evaluate the effects of anti-inflammatory agents [1].

Nitric oxide is a multi-functional mediator that plays an important role in cellular signaling and a variety of physiological functions in many cells and tissues, including the brain, the vasculature, and the immune system [27]. Evidence indicates that the overproduction of NO is a significant contributor to inflammatory processes and may provide an indicator of the degree of inflammation [28,29]. Therefore, the inhibition of NO overproduction is a useful measure for assessing the anti-inflammatory effects of drugs [30].

Inflammation is characterized by the production of a wide variety of free radicals, nitrogen reactive species, and cytokines—such as TNF-α, IL-6, and IL-1β—which act as modulators throughout the inflammation process [31]. TNF-α stimulates the production of other cytokines such as IL-6 and IL-1β. IL-6 is a multifunctional cytokine with pro- and anti-inflammatory properties that plays a central role in immune and inflammatory responses [32]. IL-1β is also a multifunctional cytokine that has been implicated in pain, fever, inflammation, and autoimmune conditions [33]. High levels of these cytokines elicit number of physiological effects including septic shock, inflammation, and cytotoxicity [2,34]. Thus, inhibiting the expression of cytokines in macrophage cells is very important during the anti-inflammatory response.

For up to 1000 years, chaga has been recognized for its medicinal properties and used widely for a plethora of illnesses in the world’s northern hemisphere. Numerous scientific reports, mostly with Asian and eastern European roots, have investigated the chemical composition and the biological activities of chaga from various geographical locations. However, no previous study has utilized chaga from the U.S. The results of this study show that different extracts of chaga sclerotia collected from Maine have significant anti-inflammatory activity in LPS-stimulated RAW 264.7 cells. The inhibitory effect occurred through a decrease in the production of the NO and a downregulation of TNF-α, IL-6, and IL-1β in RAW 264.7 macrophages, with no effect on cell viability at a concentration range of (50–150 μg/mL). It is especially interesting that the results detailed in this study also show that phenolic extracts obtained from different extraction methods have different anti-inflammatory properties. All extracts produced from the accelerated solvent extraction method and most conventional methods significantly reduce the level of NO, while the ME extract had no effect on the NO production at any concentration. The inhibitory effect of chaga extracts can likely be attributed to the content of phenolic compounds and resulting free-radical scavenging activity. Phenolic compounds have been widely recognized as natural molecules with potential antioxidant activity. It is well known that oxidative stress can activate a variety of inflammatory mediators that contribute to the inflammation process. Such oxidative stress can be inhibited by compounds with high antioxidant activity such as phenolic compounds. Previous studies have also reported that alcohol extracts of chaga are rich in polyphenolic compounds that possess strong antioxidant activity and can protect cells against oxidative damage. Such extracts have been reported to attenuate inflammation reactions and decrease the production of inflammatory mediators in 264.7 macrophages.

Earlier research focused on small phenolic compounds as a main constituent of alcoholic extracts of chaga and noted significant contribution to the antioxidant activity. However, no reports have examined the anti-inflammatory effect of these constituents using RAW 264.7 cells. In our previous work [22] we increased the extraction of phenolic acids from chaga by optimizing an ASE extraction method. In the current study, we investigated the NO-inhibitory activities of small phenolic compounds on stimulated RAW 264.7 cells. Results show phenolic acids influence different inflammatory mediators; some of the compounds tested did not affect the production of NO or the expression of the inflammatory cytokines, while others had a significant effect. For example, both VA and SA did not alter the production of NO and the expression of TNF-α, IL-6, and IL-1β at any of the examined concentrations. However, PA, PCA, and CA significantly reduced the production of NO and attenuated the expression of TNF-α, IL-6, and IL-1β at all the examined concentrations. This is in agreement with previous studies, which demonstrated anti-inflammatory properties for PA, PCA, and CA [35]. However, other research suggests that phenolic compounds with only one phenol ring, such as the tested compounds, generate a lesser anti-inflammatory effect, through inhibition of cytokine production; it has been hypothesized that other mechanisms may be involved in the anti-inflammatory action of phenolics [36]. Our work suggests that some of the small phenolic compounds present in chaga play a vital role in anti-inflammatory activity.

Previous evidence demonstrates that polysaccharides from many sources have a variety of therapeutic effects, including antioxidant and anti-inflammatory activities. Polysaccharides in chaga from Russia, China, and South Korea have been reported to act as immune-modulators and possess anti-inflammation properties from in vivo and in vitro studies. In this study, we examined the chemical structure of crude polysaccharides extracted from chaga tea, in both powder and bagged form, collected from Maine, USA. We evaluated the anti-inflammation effects of these extracts using LPS-stimulated RAW 264.7 cells. The results indicate that more components of chaga could be extracted from the powder form than from bagged form. For example, P6 yielded 30% polysaccharide containing 17.56% carbohydrate, 10.23% protein, 4.12% uronic acid, and 17.61% phenolic content; while B6 yielded 17.33% polysaccharide containing 11.81% carbohydrate, 7.23% protein, 2.84% uronic acid, and 8.33% phenolic content. Our data are in agreement with previous reports suggesting that more bioactive constituents can be extracted from different raw sources in powder forms than other forms. We attribute the higher extraction efficiency to the greater surface area of powdered form, which allows better penetration of the solvents to target analytes and thus higher extraction efficiency. The results show that the crude polysaccharides of chaga extracts in both forms have high phenolic content, indicating that these phenolic compounds are bound to macromolecules in chaga such as polysaccharides and melanin. Chaga contains high amounts of the water-soluble macromolecule pigments known as melanin. The dark color of the extracts suggests that a relatively high amount of melanin is present. Other reports also suggested that crude polysaccharide extract from chaga has melanin and melanin-associated phenolic compounds.

Our work demonstrates that crude polysaccharides obtained from both the powder and the bagged form significantly inhibit LPS-induced NO production in RAW 264.7 cells. We observed a higher NO inhibitory effect of polysaccharides obtained from chaga tea in the powdered form, in comparison to those in bagged form at the same concentration. Polysaccharides obtained from the powdered form significantly inhibited the production of TNF-α and IL-6; however, none of the crude polysaccharide extracts obtained in either form altered the expression of IL-1β. However, since the extract is crude, it contains high values of phenolic compounds and melanin, which may contribute to the anti-inflammatory effect of the extracts.

## 4. Materials and Methods

### 4.1. Fungal Material

Chaga sclerotia of various sizes and ages were collected from yellow birch (*Betula alleghaniensis*) in Maine forests. Removing chaga from tree surfaces with a hatchet or hammer, samples were collected by volunteers throughout the year and transported to the University of Maine Analytical Food Chemistry Laboratory where the entire sclerotia were immediately frozen and lyophilized (Model 7754511, Labconco Corporation, Kansas City, MO, USA) and ground using an electric food processor (Nutribullet, model-NBR-1201M, Los Angeles, CA, USA). The resulting powder was passed through a 20-mesh (0.84 mm) sieve and only particles with a diameter smaller than 0.84 mm (20-mesh) were collected. All processed samples were stored at 20 °C. A standard sample for all experimental work was created by combining and thoroughly mixing equal weights of individual processed powdered chaga. All sample extractions and assays were performed in triplicate.

### 4.2. Reagents

Folin–Ciocalteu (FC) reagent, 1,1-diphenyl-2-picrylhydrazyl (DPPH), 3,4-dihydroxybenzoic acid, caffeic acid, syringic acid, 3,4-dihydroxybenzaldehyde, bovine serum albumin (BSA), galacturonic acid, Griess reagent, 3-(4,5-dimethylthiazol-2-yl)-2,5-diphenyl-tetrazolium bromide (MTT), dimethyl sulfoxide (DMSO), and *Escherichia coli* LPS were purchased from Sigma–Aldrich (St. Louis, MO, USA). Ethanol, sodium carbonate, vanillic acid, diatomaceous earth, and Ottawa sand were purchased from Fisher Scientific (Fair Lawn, NJ, USA). Ultrapure water was obtained from a Millipore water system (EMD Millipore, Billerica, MA, USA). The murine macrophage (RAW 264.7) cell line, Dulbecco’s modified media (DMEM), heat-inactivated fetal bovine serum (FBS), and penicillin-streptomycin were obtained from Gibco Life Technologies. For the enzyme-linked immunosorbent assay (ELISA) the TNF-α, IL-6, and IL-1β ELISA kits were obtained from e-Bioscience, Inc. (Cincinnati, OH, USA). Cytokine ELISA kits were obtained from R&D Systems (Minneapolis, MN, USA). All reagents and solvents were HPLC or analytical grade.

### 4.3. Preparation of Polysaccharide Extracts

Chaga samples (1.5 g) placed in teabags (B) or in powder form (P) were infused (steeped) in 200 mL of boiled distilled water at 100 °C (for 3, 6, and 10 min). The infusions were filtered through sterilized gauze. Four volumes of cold 95% ethanol were added to the aqueous extract after concentrating to 30% of the original volume with rotary evaporator under reduced pressure at 60 °C. The extracts were kept at 4 °C overnight to isolate the crude polysaccharides. The precipitate was recovered by centrifugation at 20 min, 2000× *g* (Rotavapor R3000, Buchi, Switzerland), washed with acetone to remove adherent sugar residue and other small molecules, and dialyzed for two days with distilled water (cut-off Mw 8000 Da). The retained portion was concentrated; deproteinated with Sevag reagent (CHCl_3_:BuOH, 4:1, *v*/*v*) for 30 min under magnetic force stirring and the procedure was repeated two times. Finally, the extracts were centrifuged to remove insoluble material and the supernatant was lyophilized in the freeze–dry apparatus for 48 h to produce the crude polysaccharide extracts from the powder form (P3, P6, and P10) and from the bagged form (B3, B6, and B10), depending on the brewing time.

### 4.4. Preparation of Phenolic Extracts

#### 4.4.1. Green Extraction, Accelerated Solvent Extraction (ASE)

ASE was performed with a Dionex (Sunnyvale, CA, USA) ASE 200 instrument with solvent controller, following the procedure described by Alhallaf et al. [22]. Briefly, a dried, ground sample of chaga (1 g) was placed in a stainless-steel extraction cell, preheated for 2 min, and extracted with 70% (*v*/*v*) aqueous ethanol or 66% (*v*/*v*) aqueous ethanol. The extractions, shown in Table 2, were performed at three temperature ranges (130 °C, 150 °C, and 170 °C) for 30 min (two cycles for every sample) at a pressure of 1500 psi. Once the extraction was complete, the suspension obtained was centrifuged (10 min, 2000× *g*) and the solvent was removed using a rotary evaporator (Rotovap R3000, Buchi, Switzerland). The resulting powders were stored at −20 °C for further experiments.

#### 4.4.2. Conventional Solvent Extraction (CSE)

##### Maceration Extraction (ME)

Chaga powder (1 g) was macerated with 25 mL of 70% (*v*/*v*) aqueous ethanol for 48 h at room temperature. After filtration through a Whatman no. 1 filter paper, the solvent was removed using a rotary evaporator (Rotovap R3000, Buchi, Switzerland). The resulting residue was then dissolved and filtered in accordance with the procedure defined in Section 4.4.1. Extraction was carried out in triplicate.

##### Reflux Extraction (RE)

Chaga powder (1 g) was mixed with 25 mL of 70% (*v*/*v*) aqueous ethanol in a round-bottom flask. The extraction mixture was then refluxed in a water bath at 70 °C for 3 h. The resulting residue was dissolved and filtered in accordance with the procedure defined in Section 4.4.1. Extraction was carried out in triplicate.

##### Soxhlet Extraction (SE)

Chaga (1 g) was continuously extracted with 500 mL of 70% (*v*/*v*) aqueous ethanol for 48 h at 70 °C in a soxhlet apparatus. The resulting residue was then dissolved and filtered in accordance with the procedure defined in Section 4.4.1. Extraction was carried out in triplicate.

### 4.5. Determination of Total Phenolic Content (TPC)

The total phenolic content (TPC) of the extracts was determined by the Folin–Ciocalteu method described by [37]. Briefly, 20 μL of supernatant was mixed with 90 μL of a 10-fold diluted Folin–Ciocalteu reagent in a 96-well microplate. After standing for 5 min at room temperature, 90 μL of 6% sodium carbonate (Na_2_CO_3_) solution was added and the mixture was incubated at room temperature for 90 min. The absorbance was measured at 750 nm in a spectrophotometric microplate reader (Bio-Tek ELx808, Winooski, VT, USA). The absorbance of the extract was compared with a gallic acid standard curve for estimating the concentration of TPC in the sample. The TPC was expressed as milligrams of gallic acid equivalent per gram of dry weight chaga (mg GAE/g DW).

### 4.6. Determination of Total Neutral Carbohydrate Contents

The carbohydrate content of the polysaccharide extracts was determined using a slightly modified phenol-sulphuric acid method reported by Masuko et al. [38]. Briefly, 1 mL of sample solution, 0.05 mL of 80% phenol, and 5 mL of concentrated sulfuric acid were mixed and shaken. Following this, the mixture was held at room temperature for 10 min and the absorbance was measured at 490 nm. The total carbohydrate content was calculated using D-glucose as a standard.

#### 4.6.1. Determination of Uronic Acid Content

The uronic acid content of the polysaccharide extracts were measured according to the Blumenkrantz method [39] using D-galacturonic acid as a standard. Briefly, 0.2 mL of sample solution and 1.2 mL of sulphuric acid/tetraborate solution were mixed and shaken. The mixture was kept at 100 °C for 5 min, 20 mL of m-hydroxydiphenyl reagent was added and the absorbance was measured within 5 min at 520 nm.

#### 4.6.2. Determination of Protein Content

The total protein content of the polysaccharide extracts was determined by the Bradford method [40] using bovine serum albumin as a standard. Ten µL of sample solution and 200 µL of Bradford reagent were mixed and the mixture was incubated at room temperature for 5 min before reading the absorbance at 595 nm.

### 4.7. Cell Culture

The RAW264.7 cell line, derived from murine macrophages, was purchased from the American Type Culture Collection (Rockville, MD, USA). The cells were maintained at 37 °C in a humidified atmosphere of 5% (*v*/*v*) CO2 in Dulbecco’s Modified Eagle’s Medium (DMEM) supplemented with glutamine (1 mM), 10% fetal bovine serum (FBS; ATCC; Manassas, VA, USA), penicillin (50 U/mL), and streptomycin (50 μg/m). Medium was changed every two days. In all experiments, cells were grown to 70–80% confluence and subjected to no more than 20 cell passages.

#### 4.7.1. Measurement of Cell Viability

Cell viability was assessed by the MTT 3-(4,5-dimethylthiazolyl-2)-2,5-diphenyltetrazolium bromide) assay. The assay is based on the ability of mitochondria in viable cells to reduce the yellow tetrazolium salt MTT to purple formazan crystals. The method was performed according to the manufacturer’s procedure [23] with some modifications. The cells were cultured in 96-well plates at a density of 1 × 10^4^ cells/well for 24 h. The cells were then treated with the samples at different concentrations (50, 100, and 150 μg/mL (chaga extract) or 25, 50, and 100 μM (vanillic acid, caffeic acid, and syringic acid) or 5, 10, and 20 μM (protocatechuic acid and protocatechuic aldehyde) for 24 h in a humidified 5% CO_2_ atmosphere at 37 °C. After the incubation period, the media was removed and 100 μL of fresh medium and 10 μL of MTT solution were added to each well, and the plate was incubated for 2 h at 37 °C. Finally, the cell culture medium was discarded, and the formazan blue formed in the cell was re-suspended in 200 μL solubilization solution. The quantity of formazan (an indicator of cell viability) is measured by recording changes in absorbance at 540 nm using a spectrophotometric microplate reader (Bio-Tek ELx808, VT, USA). Of note, extracts and standards were dissolved in 0.05% DMSO. Cells treated with 0.05% DMSO was used as control and cells were treated with 2μM gallic acid was used as positive control. All experiments were performed in triplicate. % Cell viability was calculated using the following equation:

(1) $$\%\;\mathrm{Cell}\;\mathrm{viability}\;=\frac{\mathrm{Absorbance}\;\mathrm{of}\;\mathrm{the}\;\mathrm{extract}}{\mathrm{Absorbance}\;\mathrm{of}\;\mathrm{the}\;\mathrm{media}}\times 100$$

#### 4.7.2. Measurement of NO Production

Inhibitory effects of chaga extracts and the pure phenolic acid standards on the production of NO in RAW 264.7 cells were evaluated using a method modified from the previously reported work of Sun et al [41]. RAW 264.7 cells in 10% FBS-DMEM (without phenol red) were seeded (at 1 × 10^5^ cells/well) in 12 well plates. Cells were incubated for 24 h at 37 °C. Cells were then treated with varying concentrations of samples (50, 100, and 150 μg/mL (chaga extract) or 25, 50, and 100 μM (vanillic acid, caffeic acid, and syringic acid) or 5, 10, and 20 μM (protocatechuic acid and protocatechuic aldehyde)) for 2 h. The cells were then treated with 1 μg/mL LPS (Sigma–Aldrich) for 24 h at 37 °C. After 24 h, 100 μL of cell culture medium was mixed with 100 μL of Griess reagent, incubated at room temperature for 15 min and the absorbance was measured at 540 nm in an ELISA microplate reader (Bio-Tek ELx808, VT, USA). The values were compared with a sodium nitrite standard curve (5–100 µM).

#### 4.7.3. Cytokine Measurement

To assess the anti-inflammatory effect of chaga extracts and the pure phenolic acid standards on the expression of TNF-α, IL-6, and IL-1β were quantified using ELISA kits (e-Bioscience, Inc., Cincinnati, OH, USA). The assays were performed according to instructions provided by the manufacturer.

### 4.8. Statistical Analysis

Statistical analyses were performed with SPSS v25 (SPSS, Chicago, IL, USA). All results were expressed as the mean ± the standard error of triplicate analysis. Statistical significance (*p* < 0.05) was determined using one-way analysis of variance for independent means, followed by Tukey’s HSD test.

## 5. Conclusions

In response to increasing interest in on the health-promoting effects of chaga (*Inonotus obliquus*), we have shown that chaga collected in Maine, USA, exhibits significant anti-inflammatory properties against LPS-activated 264.7 RAW macrophages. Results suggest that extracts produced from accelerated solvent extraction and traditional aqueous chaga tea extraction of chaga powder are superior to other conventional methods (maceration, soxhlet, and reflux) when using NO production and the expression of TNF-α, IL-6, and IL-1β as measurements of anti-inflammatory potential. Although not quantified in any of extracts, we tested individual phenolic acids reported in similar extracts from numerous other studies on the RAW264.7 cell model and noted strong anti-inflammatory responses from caffeic acid (CA), protocatechuic acid (PA) and protocatechuic aldehyde (PCA), but not vanillic (VA) or syringic acids (SA). Also of interest is that powdered chaga extracted without incorporation into a traditional teabag resulted in higher total phenolic and carbohydrate content and produced a higher anti-inflammatory response on the cell model, with P6, the powdered form steeped at 100 °C for six minutes, exhibiting the highest response. Carbohydrates found in the aqueous tea extracts may also contribute to the anti-inflammatory response. Ongoing research is focused on the further development of methodology to maximize phenolic and carbohydrate content and includes identification and quantitation of individual phenolic acids and carbohydrates in chaga extracts in support of the development of promising new anti-inflammatory supplements.

## Figures and Tables

**Table 1:** Major chemical content of the crude polysaccharide extracts from chaga sclerotia. The data represent the mean ± SD of triplicate experiments.

Sample	Yield%	Carbohydrate%	Protein%	Uronic Acid%	TPC%
P3	30.66 ± 0.05 ^a^	17.02 ± 0.01 ^a^	10.53 ±1.66 ^a^	4.11 ± 0.47 ^a^	16.77 ± 0.13 ^ab^
P6	30.21 ± 0.01 ^a^	17.56 ± 0.01 ^a^	10.23 ±1.02 ^a^	4.12 ± 0.44 ^a^	17.61 ± 0.05 ^a^
P10	31.33 ± 0.04 ^a^	18.31 ± 0.05 ^a^	11.02 ±1.28 ^a^	4.11± 0.26 ^a^	17.04 ± 0.03 ^a^
B3	15.53 ± 0.01 ^c^	10.26 ± 0.08 ^b^	7.14 ± 1.83 ^b^	3.01 ± 0.53 ^b^	6.49 ± 0.04 ^d^
B6	17.33 ± 0.03 ^b^	11.56 ± 0.01 ^b^	7.11 ± 0.5 ^b^	2.57 ± 0.71 ^b^	8.33 ± 0.05 ^c^
B10	17.33 ± 0.02 ^b^	11.81 ± 0.04 ^b^	7.23 ± 2.01 ^b^	2.84 ± 1.22 ^b^	8.33 ± 0.06 ^c^

P: powdered chaga; B: bagged powdered chaga steeped at 100 °C for 3, 6, or 10 min. Different letters (a,b,c,d) in the same column denote statistically different means (*p* < 0.05) according to ANOVA and Tukey’s test% (*w*/*w*).

**Table 2:** Extraction conditions of chaga using accelerated solvent extraction ASE.

Extraction Conditions		Extract
Temperature °C	ETOH%	
170	66	ASE1
150	70	ASE2
130	70	ASE3

## Data Availability

The data presented in this study are available on request from the corresponding author.
